# Absorption and photoconductivity spectra of amorphous multilayer structures

**DOI:** 10.3762/bjnano.11.158

**Published:** 2020-11-20

**Authors:** Oxana Iaseniuc, Mihail Iovu

**Affiliations:** 1 Institute of Applied Physics, No. 5 Academiei Str., Chisinau, MD-2028, R. Moldova

**Keywords:** amorphous multilayer structures, photocurrent, transmission spectra

## Abstract

The experimental results regarding optical absorption and steady-state photoconductivity of amorphous single-layer structures (Al–As_0.40_S_0.30_Se_0.30_–Al, Al–Ge_0.09_As_0.09_Se_0.82_–Al, and Al–Ge_0.30_As_0.04_S_0.66_–Al) and of an amorphous heterostructure (Al–As_0.40_S_0.30_Se_0.30_/Ge_0.09_As_0.09_Se_0.82_/Ge_0.30_As_0.04_S_0.66_–Al) at different values of the voltage, with positive or negative polarity, applied to the illuminated top Al electrode are presented and discussed. The complex structure of the photocurrent spectra is attributed to the different values of the optical bandgap of the involved amorphous layers (*E*_g_ ≈ 2.0 eV for As_0.40_S_0.30_Se_0.30_ and Ge_0.09_As_0.09_Se_0.82_ and *E*_g_ ≈ 3.0 eV for Ge_0.30_As_0.04_S_0.66_). The obtained experimental results are discussed taking into account the light absorption depending on the nature and the thickness of each amorphous layer, on the wavelength, and on contact phenomena at the interfaces between different layers and between the amorphous layers and the metal electrodes with different work functions.

## Introduction

The As–S–Se, Ge–As–Se, and Ge–As–S ternary glass systems currently attract a lot of attention because of their wide application in IR optics, non-linear optics, photonics, optoelectronics, and as recording media for holography and e-beam lithography [1–3]. The physical properties of covalently bonded glasses are determined by the mean coordination number *Z* (average number of covalent bonds per atom) [4]. It is well known that the functionality of many photonic and optoelectronic devices is based on the intrinsic photoelectric effect. The photocurrent spectra and the kinetics of the photocurrent can provide information regarding the mechanisms of generation, recombination, and drift processes of non-equilibrium carriers in amorphous materials. Thus, investigations of stationary and transient characteristics of the photoconductivity of ternary amorphous thin films are of special interest. For thermally deposited amorphous films, the structure of which exhibits a higher level of disorder than that of the bulk glasses, the incorporation of impurity atoms is easier, and in many cases the metal additives could become electrically active. The influence of Sn impurities on stationary and transient photoconductivity was demonstrated for amorphous As_2_Se_3_Sn*_x_* thin films [5]. The introduction of Sn in the host material increases the drift mobility and the photosensitivity of the amorphous material. According to ^119^Sn Mössbauer spectroscopy studies of the As_2_Se_3_:Sn*_x_* glassy system [6] and X-ray photoelectron spectroscopy studies of As*_x_*Ge*_x_*Se_1−2_*_x_* glasses [4], the introduction of elements such as Sn or Ge in glasses based on arsenic selenides, leads to the formation of new tetrahedral Sn(Se_1/2_)_4_ and quasi-octahedral SnSe structural units or of GeSe_4_, respectively. Recently, experimental results of steady-state and transient photocurrents of amorphous Ge*_x_*As*_x_*Se_1−2_*_x_* and (As_4_S_3_Se_3_)_1−_*_x_*Sn*_x_* thin films were presented [7–8]. It was established that the predominant mechanisms of recombination of the photo-excited carriers in the investigated amorphous materials are mono- and bimolecular and that the transport is controlled through multiple trapping processes with exponential distribution of the localized states in the bandgap. Regarding the physics and applications of chalcogenide materials, multilayered amorphous thin-film structures are especially interesting because they offer advantages over single-layer structures [9–10].

## Experimental

The bulk chalcogenide glasses As_0.40_S_0.30_Se_0.30_, Ge_0.09_As_0.09_Se_0.82_, and Ge_0.30_As_0.04_S_0.66_ were prepared from the elements (Ge, As, S, and Se; 99.9999% purity) by conventional melt quenching. The initial materials were weighed and filled into quartz ampoules, which were sealed under vacuum (*P* = 10^−5^ Torr). After sealing, the ampoules were heated for 48 h at a temperature of *T* = 1000 °C. The ampoules were then let to cool in air to room temperature. Some of the glass samples were then cut and polished for optical measurements; other samples were prepared as granules of small dimensions for vacuum evaporation. For optical and photoelectric measurements, thin film samples of each amorphous material, with thickness values of *d* = 1000 nm for As_0.40_S_0.30_Se_0.30_, *d* = 500 nm for Ge_0.09_As_0.09_Se_0.82_, and *d* = 200 nm for Ge_0.30_As_0.04_S_0.66_, without and with Al electrodes, were prepared by thermal evaporation in vacuum (*P* = 10^−5^ Torr) of the synthesized initial glasses onto glass substrates. Longitudinal, instead of lateral, conductivity measurements were carried out. The thickness of each layer was chosen such that the distribution of the applied electrical field was as uniformly as possible in each layer. The transparence of the top Al electrode was 60–70%, and the sample area was *S* = 0.5 cm^2^.

In the same technological cycle, the multilayer heterostructure (HS) Al–As_0.40_S_0.30_Se_0.30_/Ge_0.09_As_0.09_Se_0.82_/Ge_0.30_As_0.04_S_0.66_–Al was prepared. The experimental samples had a sandwich configuration with two Al electrodes, of which the top electrode was transparent to the incident light. The dark conductivity σ_d_ and the spectral distribution of the stationary photocurrent *I*_ph_ = *f*(λ) have been measured under constant-current conditions using a spectrophotometer SPM-2 and an electrometrical amplifier U1-7, with a measurement error below ±1.0%. All experiments were performed at room temperature (*T* ≈ 20 °C).

## Results and Discussion

**Figure 1:**
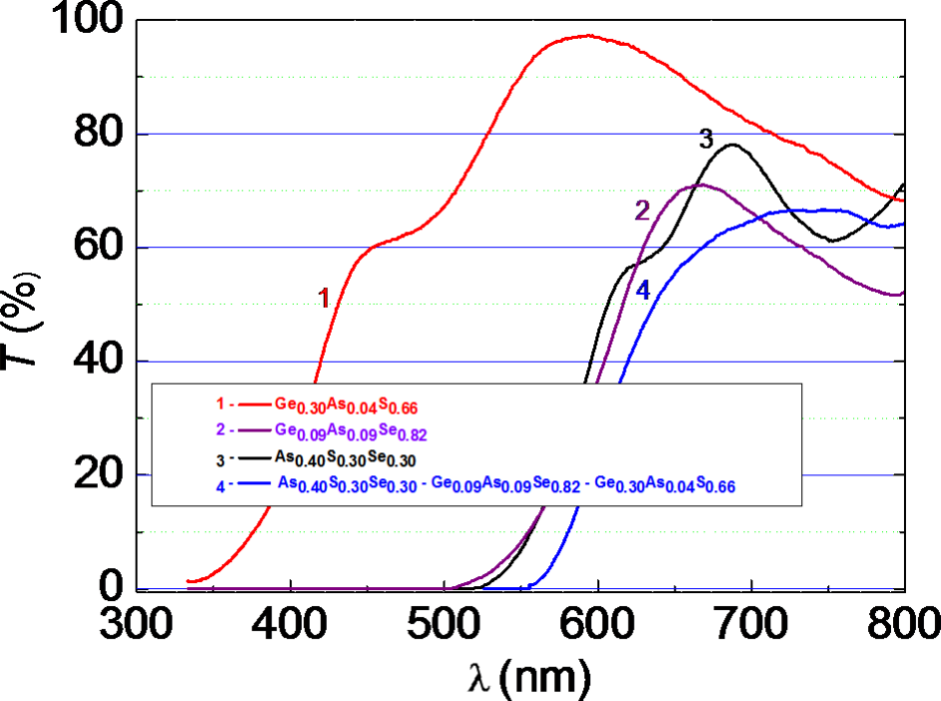
Transmission spectra *T = f*(λ) of separate amorphous thin films and multilayer HS (1: Ge_0.30_As_0.04_S_0.66_, 2: Ge_0.09_As_0.09_Se_0.82_, 3: As_0.40_S_0.30_Se_0.30_, 4: As_0.40_S_0.30_Se_0.30_/Ge_0.09_As_0.09_Se_0.82_/Ge_0.30_As_0.04_S_0.66_).

**Figure 2:**
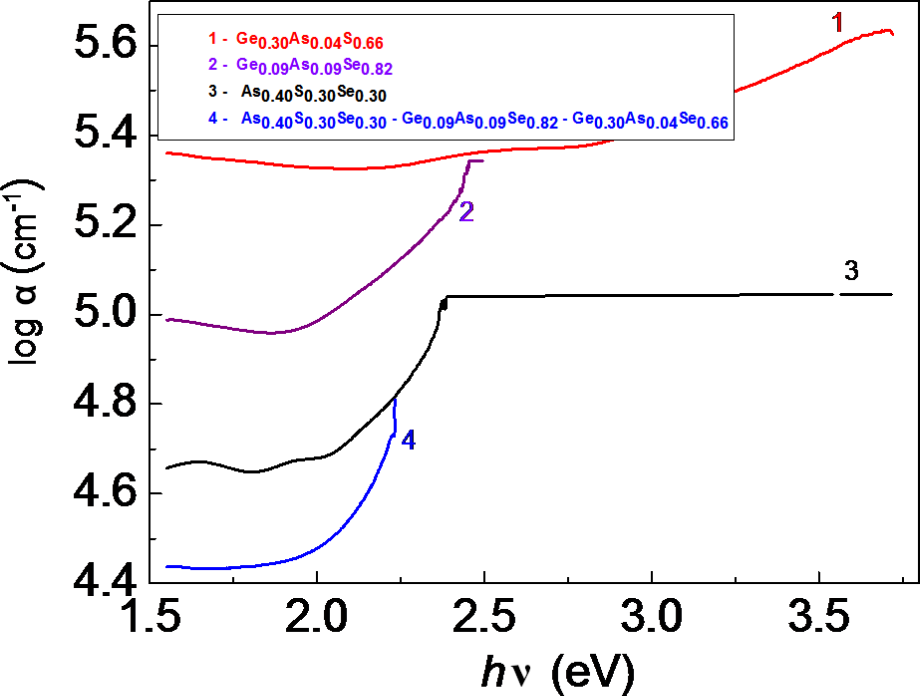
Absorption spectra α = *f*(*h*ν) of separate amorphous thin films and multilayer HS (1: Ge_0.30_As_0.04_S_0.66_, 2: Ge_0.09_As_0.09_Se_0.82_, 3: As_0.40_S_0.30_Se_0.30_, 4: As_0.40_S_0.30_Se_0.30_/Ge_0.09_As_0.09_Se_0.82_/Ge_0.30_As_0.04_S_0.66_).

Figure 1 shows the transmission spectra *T = f*(λ) of the separate amorphous thin films Ge_0.30_As_0.04_S_0.66_ (1), Ge_0.09_As_0.09_Se_0.82_ (2), As_0.40_S_0.30_Se_0.30_ (3), and the HS As_0.40_S_0.30_Se_0.30_/Ge_0.09_As_0.09_Se_0.82_/Ge_0.30_As_0.04_S_0.66_ (4). The thin film layer Ge_0.30_As_0.04_S_0.66_ with the largest bandgap energy, *E*_g_ ≈ 3.0 eV [11], which was placed on the top of the multilayer structure, has a thickness of *d* ≈ 200 nm and was transparent to the incident visible light to reach the other layers with a bandgap energy of *E*_g_ ≈ 2.0 eV [12–13] and with a thicknesses of *d* ≈ 500 nm for Ge_0.09_As_0.09_Se_0.82_ and *d* ≈ 1000 nm for As_0.40_S_0.30_Se_0.30_. Figure 2 shows that the amorphous film Ge_0.30_As_0.04_S_0.66_ is highly transparent to incident light in the visible region, in contrast to the other thin-film structures (curve 4).

Figure 2 shows the function log α = *f*(*h*ν) of the absorption coefficient for single amorphous layers and for the amorphous HS. The absorption coefficient α for each layer can be calculated using the expression:

[1]
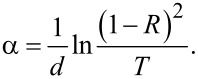


The absorption coefficient α of each separate component layer is higher than that of the HS. The highest value of the absorption coefficient α was obtained for the amorphous film of Ge_0.30_As_0.04_S_0.66_ with the smallest thickness of *d* ≈ 200 nm. The amorphous structures described above with two Al electrodes, of which the upper one was transparent to the incident light, have been used for electrical and photoelectrical measurements.

**Figure 3:**
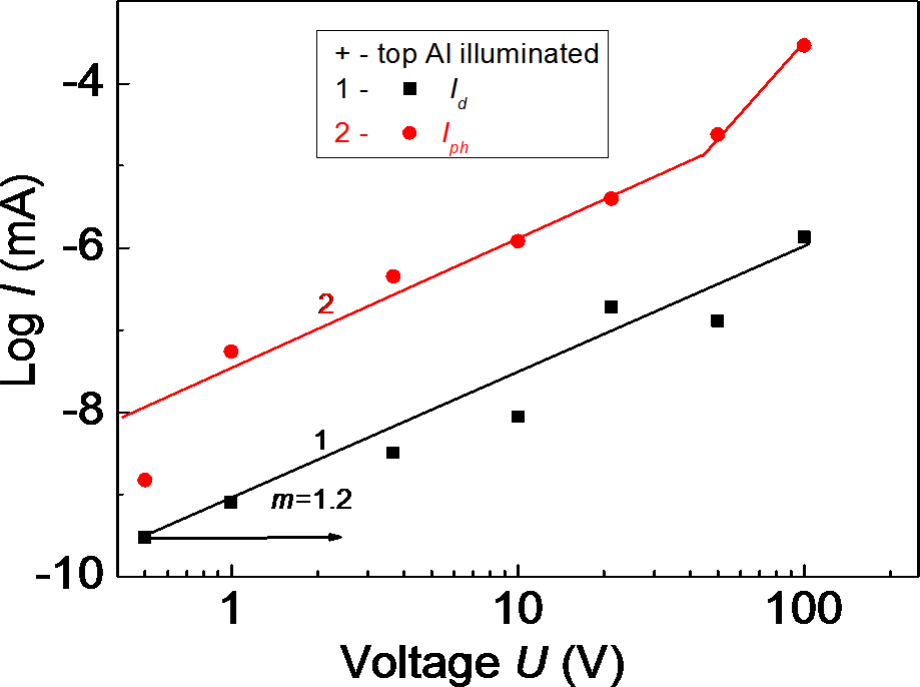
Current–voltage characteristics of the amorphous thin-film HS with a positive voltage applied to the top Al electrode, in the dark (1) and under illumination (2).

**Figure 4:**
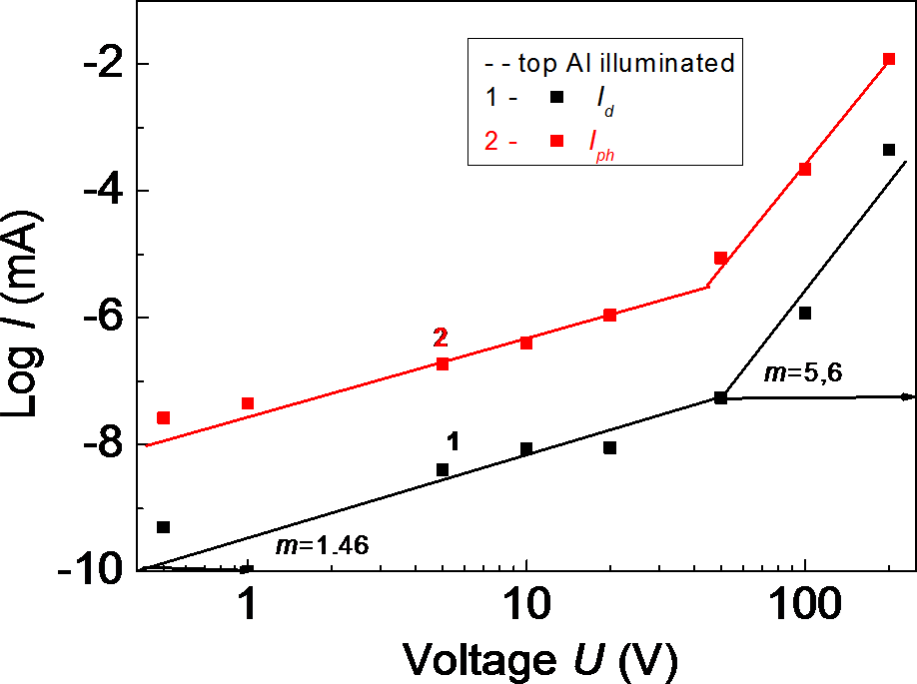
Current–voltage characteristics of the amorphous thin-film HS with a negative voltage applied to the top Al electrode, in the dark (1) and under illumination (2).

Figure 3 and Figure 4 show the current–voltage (*I–V*) characteristics of the amorphous thin-film HS Al–As_0.40_S_0.30_Se_0.30_/Ge_0.09_As_0.09_Se_0.82_/Ge_.0.30_As_0.04_S_0.66_–Al with positive or negative voltage applied to the top Al electrode in the dark (curve 1) and under illumination with the wavelength of the maximum value of the photoconductivity (curve 2).

When a positive voltage is applied to the top Al electrode, a sharp increase of the current occurs, only under illumination, when the applied external electric field reaches high values. When a negative voltage is applied, this sharp increase of the current occurs in dark and under illumination. This effect was observed for amorphous thin-film structures with different electrodes (As_2_S_3_, As_2_S_3_Ge*_x_*) and amorphous HS (As_2_S_3_/Sb_2_S_3_, Si/As_2_S_3_) [14]. *I*–*V* characteristics are described by the expression *I* = *A*·*V^m^*, where *A* is a constant coefficient, and *m* is the exponent. When carriers are injected from an amorphous or crystalline layer (or electrode) into another amorphous layer, *m* cannot take values larger than 1. The part of the *I–V* characteristics with *m* = 2 is explained by the existence of an exponential distribution of the localized sates in the bandgap of the amorphous material. In this case the *I*–*V* characteristics are described by the expression [15]:

[2]
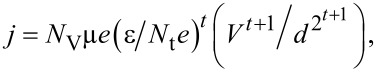


where *j* is the current density, *N*_v_ is the effective density of states in the valence band, *N*_t_ is the concentration of traps, *e* is the electron charge, µ is the drift mobility, ε is the dielectric constant, *t* = Δ/(*k*_B_*T*) (Δ is the parameter of the trap distribution, *k*_B_ is the Boltzmann constant, *T* is the absolute temperature), *V* is the applied voltage, and *d* is the sample thickness.

The explanation of our experimental data, as in the case for CIS-based solar cells with 1 < *m* < 2 (the transition part to *m* > 2) [16], is more complicated than that of the data of single-layer structures, because according to [16] the origin of the non-linear behavior may be the interface between the absorber and the buffer layer, and can thus not be attributed to space-charge-limited current (SCLC). We thank for fruitful discussion and we suggest that for more detailed information regarding the origin of SCLC in our investigated structures, additional investigations of the *I*–*V* characteristics at different temperatures are needed for the estimation of the parameters of localized states.

The steady-state photoconductivity spectra of all amorphous thin-film structures were measured with an applied external electric field of *E* = 5 × 10^4^ V/cm, that is, in the region were the *I*–*V* characteristics exhibit linear behavior. In our previous papers [7–8], we have presented the experimental results of steady-state and transient photocurrents of amorphous Ge*_x_*As*_x_*Se_1−2_*_x_* and (As_4_S_3_Se_3_)_1−_*_x_*Sn*_x_* thin films. It was demonstrated that the dependence of the steady-state photocurrent on the light intensity for all investigated amorphous thin films is non-linear, and can be described by the expression *I*_ph_ ~ *F*^α^ with the parameter 1.0 ≤ α ≤ 0.5, which indicates the existence of mono- and bimolecular recombination mechanisms in these materials.

**Figure 5:**
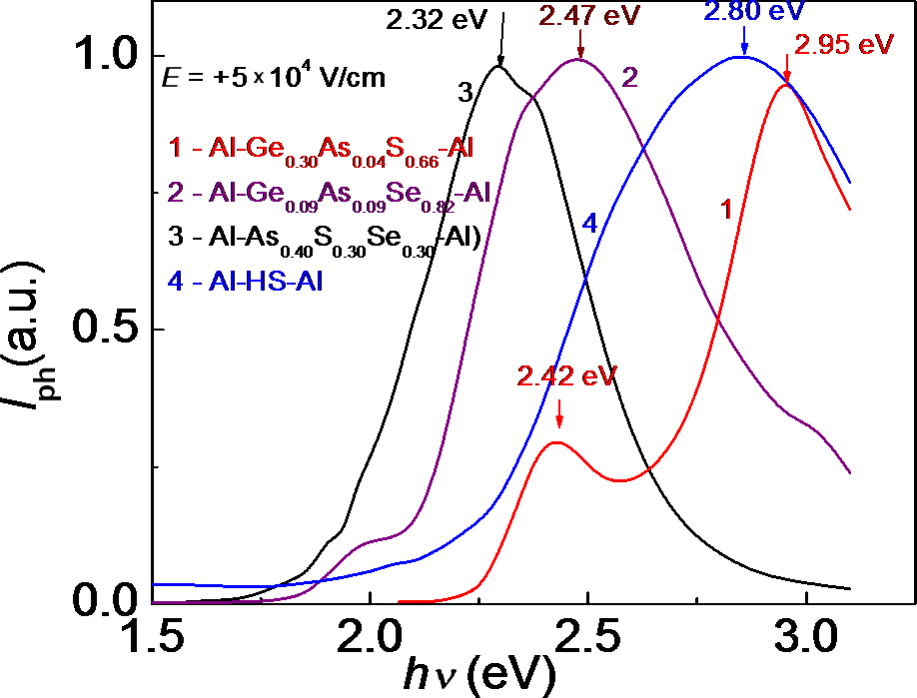
Photocurrent spectra of separate amorphous thin films and the multilayer HS with a positive voltage applied to the illuminated top Al electrode.

**Figure 6:**
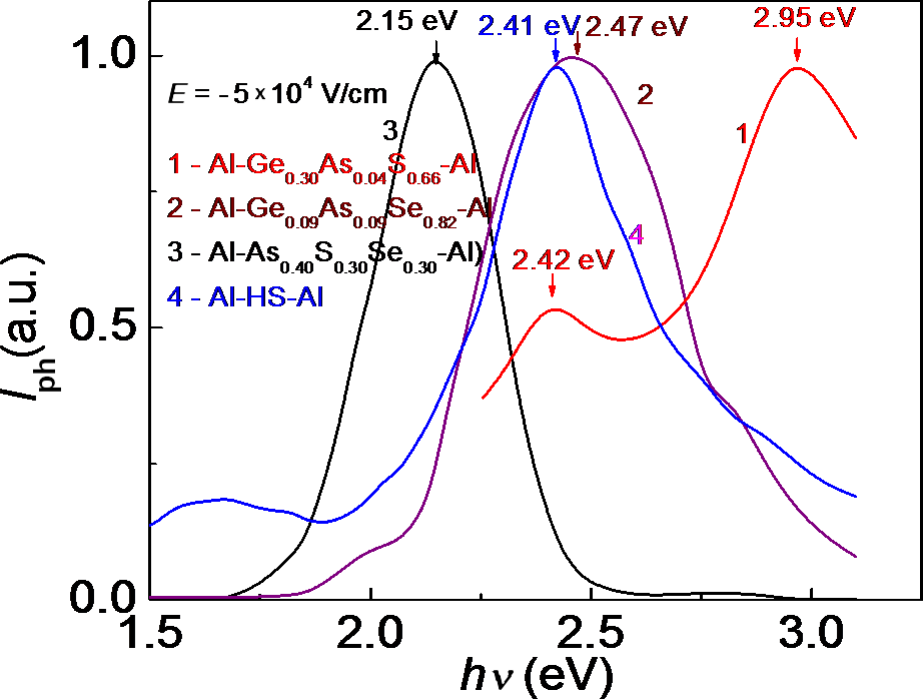
Photocurrent spectra of separate amorphous thin films and the HS with a negative voltage applied to the illuminated top Al electrode.

Figure 5 and Figure 6 show the steady-state photocurrent spectra of separate amorphous thin films and HS Al–As_0.40_S_0.30_Se_0.30_/Ge_0.09_As_0.09_Se_0.82_/Ge_.0.30_As_0.04_S_0.66_–Al with positive or negative voltage applied to the illuminated top Al electrode. The steady-state photocurrent spectra show that the maximum of photosensitivity of the component layers of the multilayer HS is in a good agreement with the absorption spectra. The spectral distribution of the photocurrent for the HS is situated between the distributions of the separate layers and depends on the polarity of the illuminated electrode. The positions of the maximum of Al–Ge_0.09_As_0.09_Se_0.82_–Al (*h*ν = 2.47 eV) and the maxima of Al–Ge_0.30_As_0.04_S_0.66_–Al (*h*ν = 2.95 eV and *h*ν = 2.42 eV) do not depend on the polarity of the applied voltage.

The presence of two maxima in the photoconductivity spectra of Al–Ge_0.30_As_0.04_S_0.66_–Al, located at *h*ν = 2.95 eV and *h*ν = 2.42 eV, indicates a probable separation of this sample into multiple phases that contain trigonal (AsS_3/2_) and tetrahedral (GeS_4_) structural units, respectively. Figure 5 and Figure 6 also show that for the thin-film structure Al–As_0.40_S_0.40_Se_0.30_–Al and for the multilayer HS the position of the maximum in the spectral distribution of the photocurrent depends on the polarity of the applied voltage. When a positive voltage is applied to the illuminated Al electrode, the maximum is shifted toward the region of higher energies of the spectrum. As was mentioned in [8], this behavior can be associated with contact phenomena at the interfaces between metallic electrode and amorphous layers, as well as with the drift and surface recombination of the non-equilibrium carriers [14].

**Figure 7:**
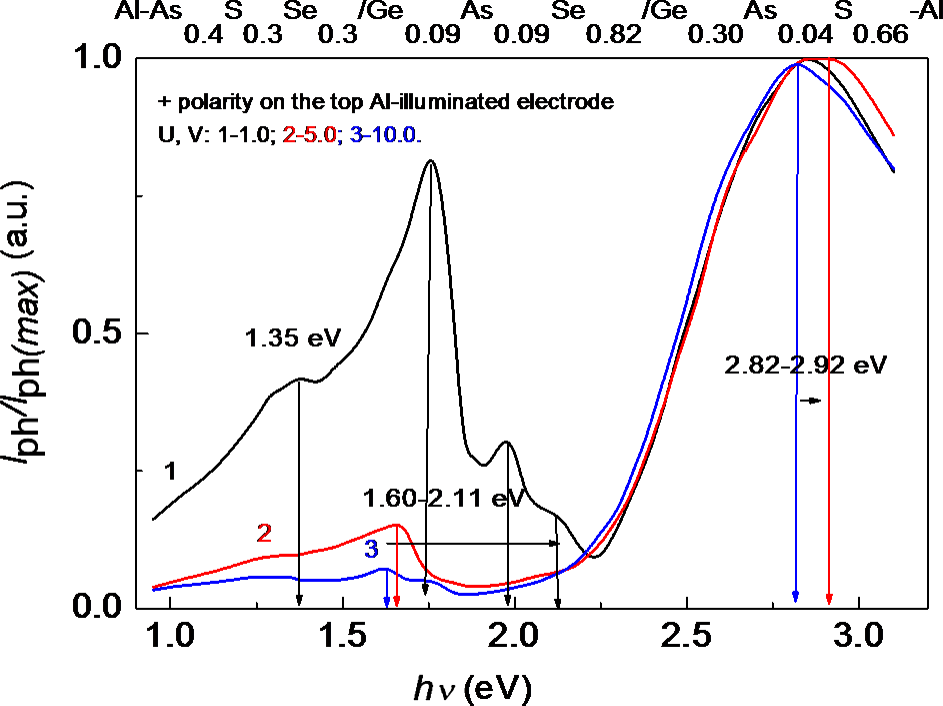
Normalized photocurrent spectra of the multilayer HS with different positive values of the voltage applied to the illuminated top Al electrode.

**Figure 8:**
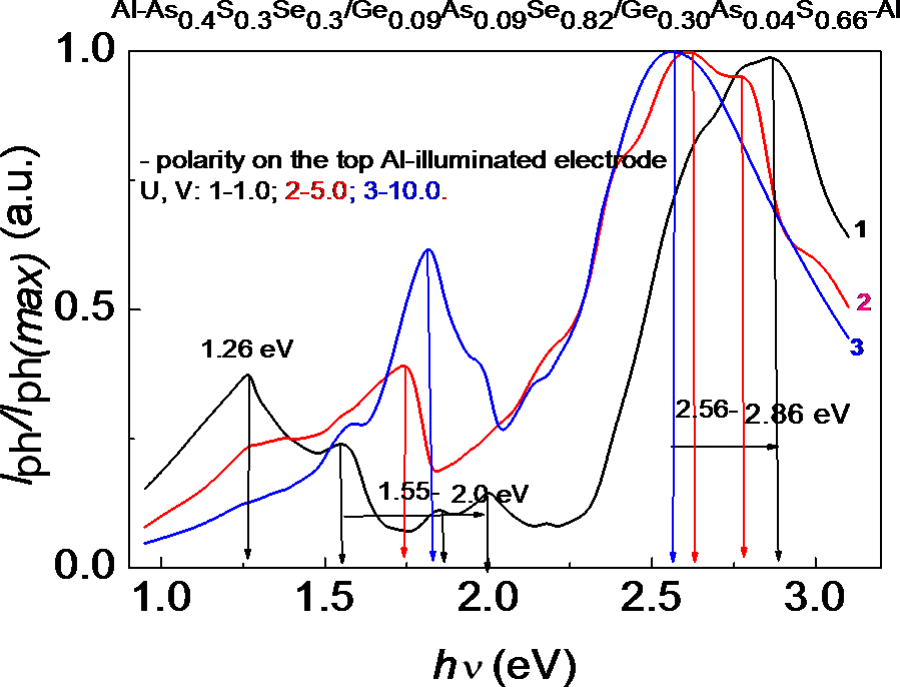
Normalized photocurrent spectra of the multilayer HS with different negative values of the voltage applied to the illuminated top Al electrode.

The normalized photocurrent spectra of the multilayer thin-film HS at different, positive and negative, values of the voltage applied to the illuminated top Al electrode (*U* = 1.0–10.0 V) are presented in Figure 7 and Figure 8. It is evident that increasing the positive applied voltage leads to a shift of the photocurrent maximum toward higher energies (Figure 7). An increase of the negative applied voltage leads to a shift of the photocurrent maximum toward lower energies (Figure 8). This shift can be attributed to the different resistance values and the different *I–V* characteristics of the component layers of the HS. The distribution of the applied voltage among the layers depends on the photocurrent. Therefore, the contribution of each layer to the total photocurrent is different. When the applied voltage has a positive polarity (0.1–200 V), the structure of the photocurrent spectra is more complicated. It was observed that at low applied voltages (0.1–0.5 V) the photocurrent is negative, and at *U* = 1.0 V the photocurrent changes to positive values. This phenomenon can occur when the external applied field becomes higher than the internal electrical field. For amorphous semiconductors the barrier height of an Al–semiconductor contact, with a work function of φ_m_ = 4.18 eV, is φ_b_ = 0.40–0.75 eV [14,17]. This is very important from a practical point of view, because there is the possibility to build photodetectors with different signs of the photocurrent. This phenomenon of photocurrent sign inversion through polarization was also observed for photodetectors based on ZnAs_2_ anisotropic crystals [18].

Figure 8 shows that, besides the main peaks in the spectral distribution of the photocurrent at 2.56–2.92 eV, there are also significant peaks in the region of 1.26–2.11 eV. When the voltage applied to the top Al electrode has a positive polarity, the maximum at 2.82–2.92 eV is attributed to the absorption of light in the wide bandgap of Ge_0.30_As_0.04_S_0.66_ (*E*_g_ ≈ 3.0 eV). It weakly depends on the applied voltage, Δ(*h*ν) = 0.1 eV. When the voltage applied to the top Al electrode has a negative polarity, the maximum at 2.56–2.86 eV is also attributed to the absorption of light in the wide bandgap of Ge_0.30_As_0.04_S_0.66_ (*E*_g_ ≈ 3.0 eV). However, it depends more strongly on the applied voltage, Δ(*h*ν) = 0.3 eV. The other maxima at 1.26–2.11 eV correspond to the narrow-bandgap materials Ge_0.30_As_0.04_S_0.66_ and Ge_0.09_As_0.09_Se_0.82_. Some of them are also present in the spectral distribution of the single-layer structures (Figure 5 and Figure 6). Spectral position and amplitude of these maxima depend, among others, on the value and polarity of the applied voltage.

**Figure 9:**
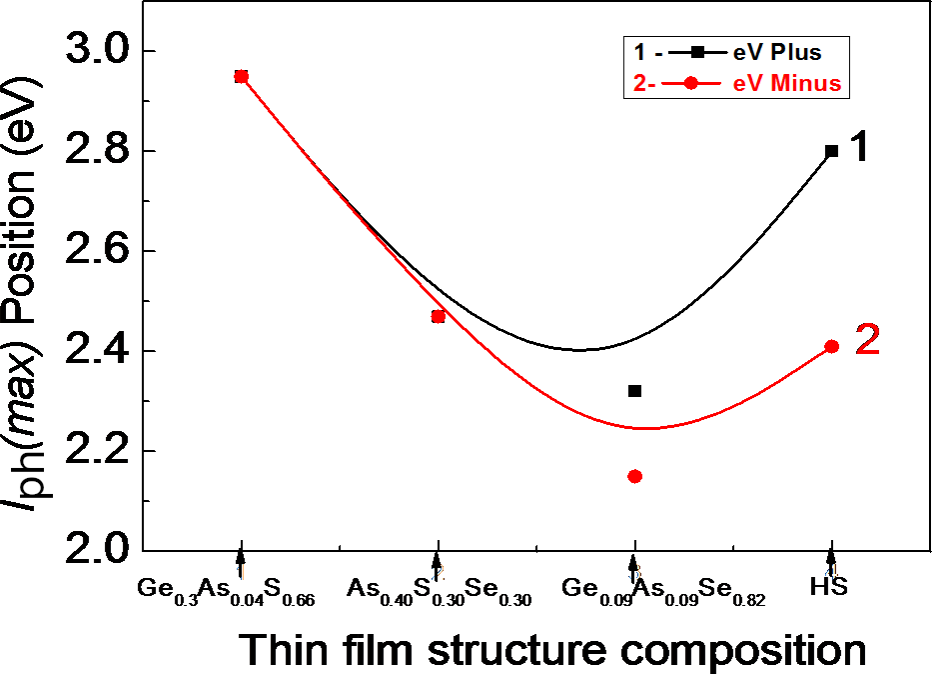
Peak position in the spectral distribution of the photocurrent for the different thin-film structures at positive (curve 1) and at negative (curve 2) polarity of the voltage applied to the illuminated top Al electrode. The lines are a guide to the eye.

Figure 9 shows the position of the photocurrent maximum of the different components of the HS and of the HS itself at positive (curve 1) and at negative (curve 2) polarity of the voltage applied to the illuminated top Al electrode. The spectral position of the maximum of the photocurrent of the HS has an intermediate value with respect to the component layers.

**Figure 10:**
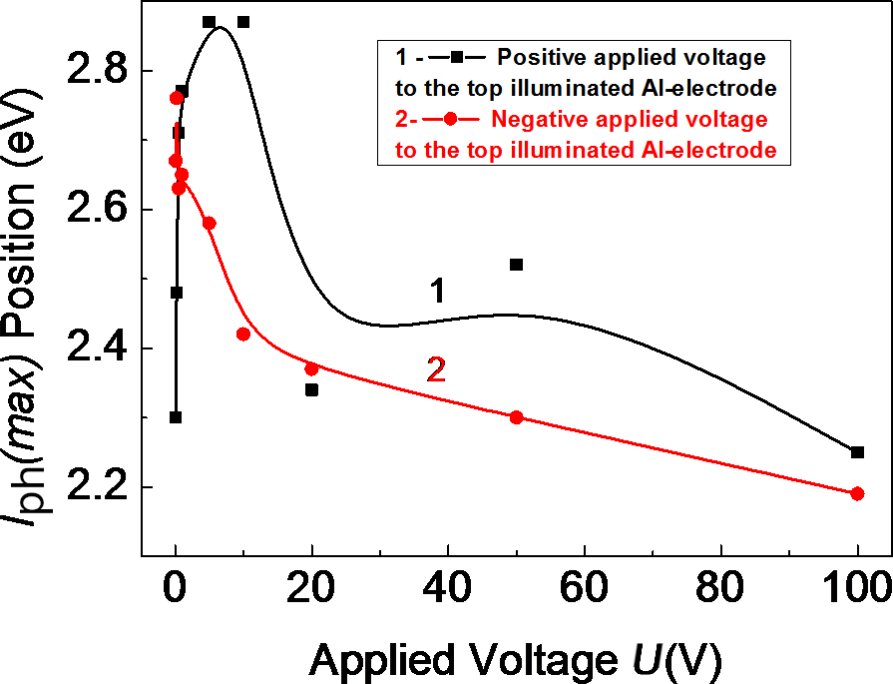
Peak position in the spectral distribution of the photocurrent of amorphous thin-film HS as a function of the applied voltage with positive (1) and negative (2) polarity (applied field *E* = 5 × 10^4^ V/cm). The lines are a guide to the eye.

Figure 10 shows the peak position in the spectral distribution of the photocurrent of the amorphous thin-film HS as a function of the applied voltage with positive (1) and with negative (2) polarity. At low applied voltages with positive polarity (*U* ≤ 10 V), a shift of the maximum of the photocurrent to higher photon energies (from 2.3 eV to 2.9 eV) is observed. When the applied voltage is increased further up to *U* = 100 V, the maximum returns to its initial position (*h*ν = 2.3 eV, curve 1). At negative polarity, the maximum of the photocurrent is shifted only to lower photon energies (from 2.75 to 2.2 eV).

**Figure 11:**
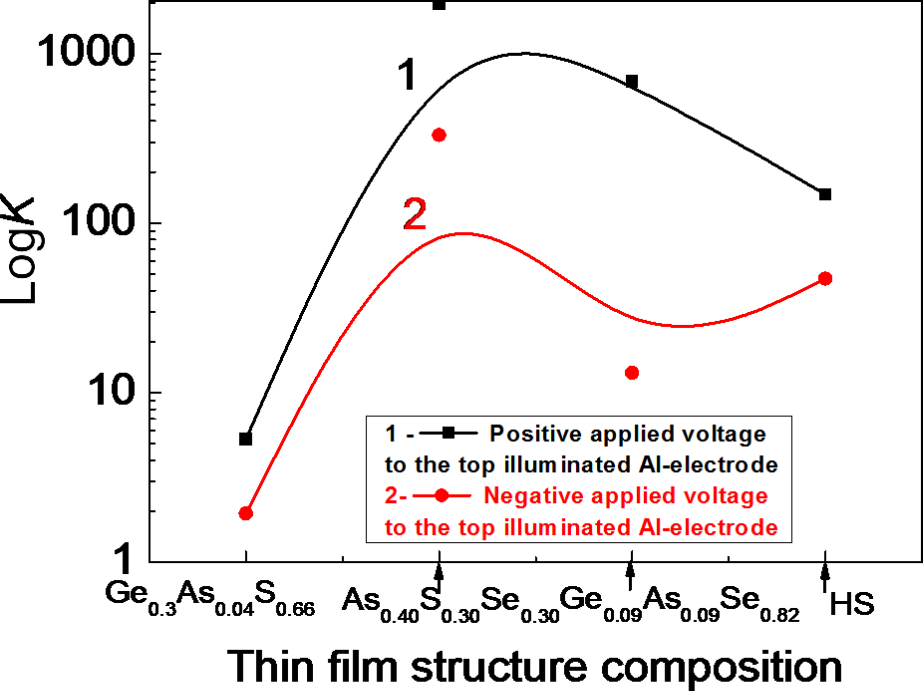
The magnifying power *K* of the different thin-film structures at positive (1) and at negative (2) polarity of the applied voltage (applied field *E* = 5 × 10^4^ V/cm). The lines are a guide to the eye.

Figure 11 shows the magnifying power *K* (i.e., the ratio between photocurrent and dark current, *K* = *I*_ph_/*I*_dark_) of the thin-film structures and the HS both at positive (curve 1) and at negative (curve 2) polarity of the applied voltage. The value of *K* is higher at positive polarity than at negative polarity. Also, the amorphous Al–As_0.40_S_0.30_Se_0.30_–Al thin-film structure shows the highest values of *K*.

**Figure 12:**
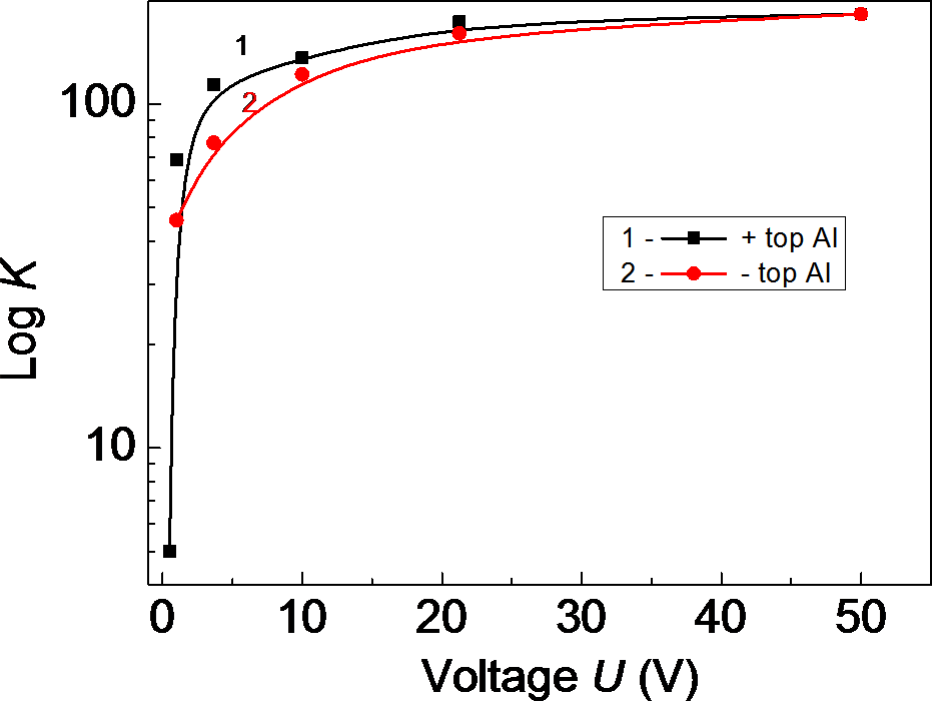
The magnifying power *K* of the amorphous thin-film HS as a function of the applied voltage with positive (1) and negative (2) polarity. The lines are a guide to the eye.

Figure 12 shows the dependence of the magnifying power *K* of the amorphous thin-film HS as a function of the applied voltage with positive (1) and with negative (2) polarity. The photosensitivity is higher when the voltage applied at the illuminated top Al electrode has a positive polarity, which was also demonstrated for structures of single thin films [7].

## Conclusion

The experimental results regarding optical absorption and steady-state photoconductivity of both amorphous single-layer structures, Al–As_0.40_S_0.30_Se_0.30_–Al, Al–Ge_0.09_As_0.09_Se_0.82_–Al, and Al–Ge_0.30_As_0.04_S_0.66_–Al and the heterostructure Al–As_0.40_S_0.30_Se_0.30_/Ge_0.09_As_0.09_Se_0.82_/Ge_0.30_As_0.04_S_0.66_–Al have been discussed. The spectral distribution of the photocurrent depends on the value and on the polarity of the voltage applied to the illuminated top Al electrode. It was found, that at low applied voltages with positive polarity (*U* ≤ 10 V) the maximum of the photocurrent is shifted to higher photon energies (from 2.3 to 2.9 eV) with increasing voltage. When the applied voltage is further increased up to *U* = 100 V, the maximum returns to its initial position.

The shift of the photocurrent maximum may be related to the component layers of the HS. Because the resistance of the wide-bandgap material (Ge_0.30_As_0.04_S_0.66_) is higher, at low voltages the electrical field mainly is distributed in this material, which leads to a photoconductivity maximum in the high-energy region. When the voltage is further increased, the electrical field is also distributed in the narrow-bandgap materials and the position of the photocurrent maximum is shifted in to the low-energy region. For GeTe and GeSe films, an analogous shift was explained by bulk phenomena in the material, not by contact phenomena [19]. In contrast, when the applied voltage has a negative polarity, the maximum of the photocurrent is shifted only to lower photon energies (from 2.75 to 2.2 eV).

Besides that, the magnifying power, *K* = *I*_ph_/*I*_dark_, depends on the composition of the thin-film structures and of the HS and on the polarity of the applied voltage as well. Moreover, it was demonstrated that higher values of the magnifying power *K* were reached at positive polarity for all investigated amorphous thin-film structures. This result can be explained by drift processes of non-equilibrium carriers in amorphous semiconductors as well as by the contact phenomena between interfaces of different amorphous materials and the metallic electrodes.
